# A Highly Birefringent Photonic Crystal Fiber for Terahertz Spectroscopic Chemical Sensing

**DOI:** 10.3390/s21051799

**Published:** 2021-03-05

**Authors:** Tianyu Yang, Liang Zhang, Yunjie Shi, Shidi Liu, Yuming Dong

**Affiliations:** 1 Shenzhen Institute of Advanced Technology, Chinese Academy of Sciences, Shenzhen 518055, China; yangtianyu150@outlook.com (T.Y.); liang.zhang@siat.ac.cn (L.Z.); yj.shi@siat.ac.cn (Y.S.); liusd@siat.ac.cn (S.L.); 2 Guangdong-Hong Kong-Macao Joint Laboratory of Human-Machine Intelligence-Synergy Systems, Shenzhen Institutes of Advanced Technology, Chinese Academy of Sciences, Shenzhen 518055, China; 3 Schools of Science, Changchun University of Science and Technology, 7089 Weixing Road, Changchun 130022, China; 4 Fiberhome Technologies College, Wuhan Research Institute of Posts and Telecommunications, Wuhan 430070, China

**Keywords:** chemical sensing, terahertz, fiber sensor, relative sensitivity

## Abstract

A photonic crystal fiber (PCF) with high relative sensitivity was designed and investigated for the detection of chemical analytes in the terahertz (THz) regime. To ease the complexity, an extremely simple cladding employing four struts is adopted, which forms a rectangular shaped core area for filling with analytes. Results of enormous simulations indicate that a minimum 87.8% relative chemical sensitivity with low confinement and effective material absorption losses can be obtained for any kind of analyte, e.g., HCN (1.26), water (1.33), ethanol (1.35), KCN (1.41), or cocaine (1.50), whose refractive index falls in the range of 1.2 to 1.5. Besides, the PCF can also achieve high birefringence (∼0.01), low and flat dispersion, a large effective modal area, and a large numerical aperture within the investigated frequency range from 0.5 to 1.5 THz. We believe that the proposed PCF can be applied to chemical sensing of liquid and THz systems requiring wide-band polarization-maintaining transmission and low attenuation.

## 1. Introduction

Mircostructured fibers (MFs) or photonic crystal fibers (PCFs) with air holes periodically arranged in the cross-section have attracted considerable attention in the last two decades due to their unusual design flexibility. Compared to the conventional step-index fibers, PCFs can yield some unique characteristics—e.g., endless single mode, tunable dispersion, large effective mode areas, high nonlinearity, high birefringence, and large light-interaction areas [1,2,3].

Since the first PCF was reported in the end of the last century [4], attention was mainly focused on its optical applications. It is important to notice, however, that it also has great potential to work at other frequency ranges, such as the terahertz (THz) range. The THz band, which covers the spectral range from 0.1 THz to 10 THz, could effectively alleviate the spectrum scarcity and capacity limitations of current systems. Unlike the microwave and optical bands, many features of the THz band are only now being studied. One important reason that the THz band was not intensively investigated before is the high transmission loss by the moisture in air during its propagation. To alleviate the loss issue of THz communications, THz optical PCFs [5] have been used as waveguides in many applications, such as THz antennas [6] and polarization-maintaining THz systems [7].

More importantly, the THz and optical fiber sensors [8] have been well developed and a number of THz PCF-based, environmentally friendly sensors have been proposed in recent years, which involve pressure sensing [9], biosensing [10], temperature sensing [11], salinity sensing [12], and chemical sensing [13]. To maximize the advantages and sensitivity responses of PCF-based sensors, many kinds of geometrical structure have been designed, such as the kagome lattice [14], the hexagonal lattice [15], the elliptical porous core [16], the D-shape [17], and the octagonal lattice [18]. In early 2008, Kiwa et al. [19] reported a pH sensor in the THz regime. To increase the pH sensitivity level, extra biasing voltage was provided. Another fiber-sensor reported in [20] was adopted to monitor the aging of the industrial coolant fluids by detecting their pH values. Md. Islam et al. [11] proposed a THz PCF-based sensor comprising a porous core and kagome cladding to detect three different liquids, i.e., water, ethanol, and benzene. The maximum relative sensitivity is 85.7% at 1.6 THz. It was indicated that the adoption of kagome cladding was to increase the refractive index contrast between the core and cladding, which in turn can increase the portion of THz waves in the core area, and thus increase the sensitivity and reduce the confinement loss. An important point, however, is that the use of kagome cladding also introduces extra complexity for fiber implementation. To reduce the complexity, later in the same year, a suspension type of cladding was used in the THz PCF-based sensor [21]. The sensor is able to detect highly toxic cyanide (CN), and the relative sensitivity can be as high as 85.8% at 2.0 THz. The reported PCF can also provide a birefringence of 0.009 and a low effective material absorption loss of 0.09 dB/cm. In [22], a relatively simple geometry was used in constructing the PCF-based sensor. The PCF had a quasi-circular shaped cladding and a porous core built only by circular air holes. The realized relative sensitivities for the targeted analytes ethanol, benzene, and water, were 78.8%, 77.8%, and 69.7% at 1.3 THz, respectively. Later, a hybrid PCF [23] including circular and elliptical air holes was reported for detecting the concentration of H${}_{2}$SO${}_{4}$ solutions. The relative sensitivity was as high as 63.4%. Apart from the analytes mentioned above, the detection of blood components, such as hemoglobin, red blood cells (RBCs), white blood cells (WBCs), and plasma was also investigated in [24]. The highest sensitivity of the PCF was 80.93% at 1.5 THz.

It was noted that all the aforementioned designs employed porous cores, which are widely used in THz PCFs, as they can reduce the effective material absorption loss (EML) [25,26,27]. However, it is not necessary to use such a core to design PCFs for chemical sensing. The main reason is that for the sensing scenario, all the air holes are filled with the analyte. Therefore, the variation of EML is not significant, whether or not a porous core is adopted. Besides, the existence of the porous core introduces more complexity. Additionally, it is noted that there is no reported PCF that is able to detect multiple analytes. In this work, a novel PCF containing a suspended rectangular shaped core is proposed, which can sense any liquid analytes with refractive indices within the range of 1.2 to 1.5. Four straight struts are employed in the cladding, producing a hollow core to be filled by the analytes. The proposed PCF not only has great sensitivity performance, but also can yield high birefringence, low and flat dispersion, low loss, a large effective mode area, and a large numerical aperture simultaneously. To the best of our knowledge, there is no alternative with those characteristics.

## 2. PCF Configuration

**Figure 1:**
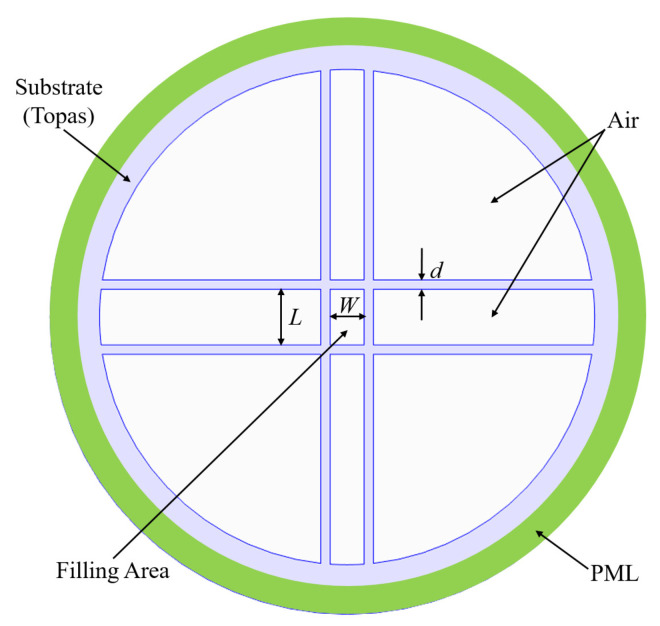
Cross-sectional view of the proposed photonic crystal fiber (PCF).

The cross-section of the proposed PCF is shown in Figure 1, where four dielectric struts are crossed in the cladding. As a result, a rectangular shaped core area is formed, which analytes can fill. The distance between the two parallel struts along the *x*-axis is *L* and the distance between the two along the *y*-axis is *W*, thus, the length and width of the rectangular core are *L* and *W*, respectively. The thickness of each strut is *d*. Topas [28] is selected as the substrate because of its outstanding properties, i.e., low bulk material absorption loss < 0.2 cm${}^{-1}$ and stable refractive index (n=1.526±0.001) below 1.5 THz. Note that the material absorption loss will increase with the increase of frequency. Particularly, the loss of Topas will be over 1 cm${}^{-1}$ after 1.5 THz. To avoid high material absorption loss, we chose 1.0 THz as the center frequency and investigated the PCF across the frequency range of 0.5 to 1.0 THz. The reason for choosing 1.0 THz as the center frequency was to increase the possibility of integrating the proposed PCF with other reported waveguiding PCFs [25,26,27,29], which are designed based on the operation frequency of 1 THz.

All the simulations in this work were conducted with commercial finite-element-method-based software—COMSOL Multiphysics simulator [30]. The PCF structure was developed with the 2-dimensional modeling function in the Radio Frequency module. The Eigenmode Solver was adopted to obtain the propagation constants of the supported modes. The mode number was first set at 30 to widely search the guided modes and to learn how many modes actually played a serious role in the calculations, which number was usually found to be about 5. Very fine meshes were chosen to obtain accurate results. Thus, the simulation worked well when about 30,000 meshes were created. The target solution resolution was set at 10${}^{-8}$ and was reached generally in five iterations. A perfectly matched layer (PML) with a thickness of 10% of the overall radius of the cladding, represented by the green ring in Figure 1, was built to absorb any radiations leaking out of the fiber. The software can directly obtain the field distributions, but to obtain loss values, internal post-processing tools are required.

Note that the aim of this work was to design a PCF that senses the analytes having refractive indices within the range from 1.2 to 1.5. Therefore, we first set the refractive index of the core area to be 1.35, the mean value of 1.2 and 1.5, in order to optimize the PCF dimensions. The optimized values of the dimensions are: *W* = 280 μm, L=Ratio∗W, Ratio = 1.5, and *d* = 10 μm. The radius of the cladding was 2000 μm and the thickness of the PML was 200 μm. Later, three analytes, i.e., HCN (*n* = 1.26) [21], ethanol (*n* = 1.35) [11], and RBCs (*n* = 1.40) [24], were selected as the representatives to further test the sensing performance of the proposed PCF-based sensor.

## 3. PCF Characteristics

To quantify the fiber efficiency as a gas or liquid sensor, one important parameter is the relative sensitivity [31].

(1) $$\begin{array}{c} R=\frac{n_{r}}{n_{eff}}\times F, \end{array}$$

where $n_{r}$ is the refractive index of the analyte filled in the fiber core, $n_{eff}$ is the modal effective index associated with the propagation constant, and *F* represents the energy portion in the core area and it can be obtained through [31]:

(2) $$\begin{array}{c} F=\frac{\int_{sample}Re\left(S_{z}\right)dxdy}{\int_{total}Re\left(S_{z}\right)dxdy}\times 100\%, \end{array}$$

$\int_{sample}$ means that the integration is taken over the area filled by the analyte and $\int_{total}$ means that the integration is taken the entire fiber cross-section. $S_{z}$ is the Poynting vector projection in the *Z* direction.

The polarization maintaining (PM) ability is usually an important factor that people consider when they investigate chemical sensors. PM PCF can eliminate the polarization modal dispersion, preserve the polarization state of the input THz wave, and increase the stability of optical devices [11]. The birefringence (*B*) of a fiber is the parameter used to evaluate its PM ability. It can be obtained from [3]

(3) $$\begin{array}{c} B=|n_{x}-n_{y}|, \end{array}$$

where $n_{x}$ and $n_{y}$ are the effective modal refractive indices for the X-polarized (XP) and Y-polarized (YP) modes, respectively.

Another important characteristic of a PCF is its propagation loss. For a PCF-based sensor, although the required length of the PCF is generally not very long, the loss is desired to be as low as possible. In this case, the loss mainly contains two parts, the confinement loss and the effective material absorption loss (EML). While the confinement loss is used to specify the leakage of the THz wave from the fiber, the EML represents the loss introduced by the substrate. They can be separately obtained by [22]

(4) $$\begin{array}{c} L_{c}\left({\mathrm{cm}}^{-1}\right)=\frac{4\pi f}{c}\times Im[n_{eff}], \end{array}$$

where *c* is the speed of light in vacuum, *f* is the operation frequency, and $Im[n_{eff}]$ is the imaginary part of the effective refractive index, and by

(5) $$\begin{array}{c} \alpha_{eff}\left({\mathrm{cm}}^{-1}\right)=\frac{{\left(\frac{\epsilon_{0}}{\mu_{0}}\right)}^{1/2}\int_{mat}n_{mat}\alpha_{mat}{\left|E\right|}^{2}dxdy}{2\int_{total}S_{z}dxdy}, \end{array}$$

where $\epsilon_{0}$ and $\mu_{0}$ are the permittivity and permeability of vacuum, $n_{mat}$ is the refractive index of the background material, $\alpha_{mat}$ is the bulk material absorption loss, and *E* is the modal electric field.

To guarantee a good communication capacity, the optical dispersion of a fiber is required to be as low and flat as possible, because a dramatic variation of the dispersion would introduce a significant performance variation across the bandwidth. Since the material dispersion of Topas is quite stable within the investigated frequency band, only the waveguide dispersion is considered here, which can be calculated by [3]:

(6) $$\begin{array}{c} \beta_{2}=\frac{2}{c}\frac{dn_{eff}}{d\omega }+\frac{\omega }{c}\frac{d^{2}n_{eff}}{d\omega^{2}}, \end{array}$$

where ω=2πf represents the central angular frequency.

The effective mode area is the area covered by the mode field. It is usually calculated by [32]

(7) $$\begin{array}{c} A_{eff}=\frac{{\left(\;\int \int \;|E\right|}^{2}{dxdy)}^{2}}{{\;\int \int \;|E|}^{4}dxdy}, \end{array}$$

where |E| is the magnitude of the electric field of the investigated mode.

A large numerical aperture (NA) is also desirable for broad band fiber sensing applications [11]. To enhance the value, a large refractive index contrast between the core and cladding is required. Due to the extremely simple cladding, the index contrast in our PCF is quite high. Consequently, the NA performance of the proposed PCF would be reasonable. The value of NA can be obtained from the following expression [22].

(8) $$\begin{array}{c} NA=\frac{1}{\sqrt{1+\frac{\pi A_{eff}f^{2}}{c^{2}}}}. \end{array}$$

## 4. Numerical Simulations and Analysis

**Figure 2:**
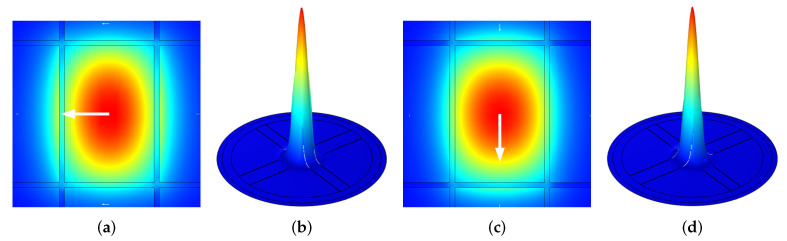
E-field distribution of the optimized PCF filled with ethanol for (**a**,**b**) XP mode and (**c**,**d**) YP mode at 1 THz. Among them, (**b**,**d**) indicate the density distributions.

As mentioned, we will firstly exhibit the results for the case where ethanol (n=1.35) is filled in the core area to demonstrate the process of developing the optimized PCF. The E-field distribution of the XP and YP modes for the optimized PCF filled with ethanol are shown in Figure 2. It can be observed that the THz wave can be well confined in the core area of both the XP and YP modes.

**Figure 3:**
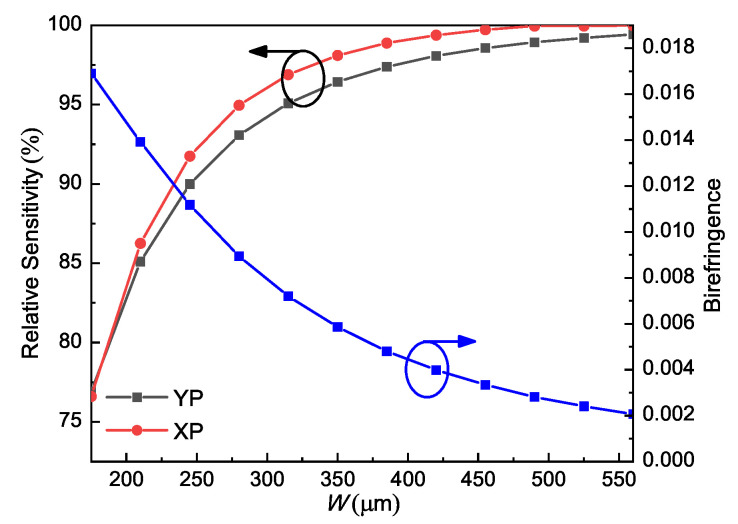
Relative sensitivity and birefringence of ethanol-filled PCF as functions of *W* at 1 THz; the rest parameters are L=Ratio∗W, Ratio = 1.5, *d* = 10 μm.

The relative sensitivity and birefringence of the ethanol-filled PCF as functions of core width *W* for XP and YP modes are shown in Figure 3; the other parameters were set as L=Ratio∗W, Ratio = 1.5, and *d* = 10 μm. It is noted that the relative sensitivities for both polarized modes increased with *W*. However, a larger core would support multiple higher order modes. For some applications involving interferometric detection or property sensing with phase coding, single-mode or several-mode propagation is required. Besides, the birefringence decreases with *W*. Considering the trade-off, *W* = 280 μm was selected in this design. The corresponding relative sensitivities were 95.1% and 93.1% for the XP and YP modes, respectively. For applications that allow multiple higher-order modes and lower birefringence, a larger core could be used. In that case, near 100% relative sensitivity of the XP mode could be obtained.

**Figure 4:**
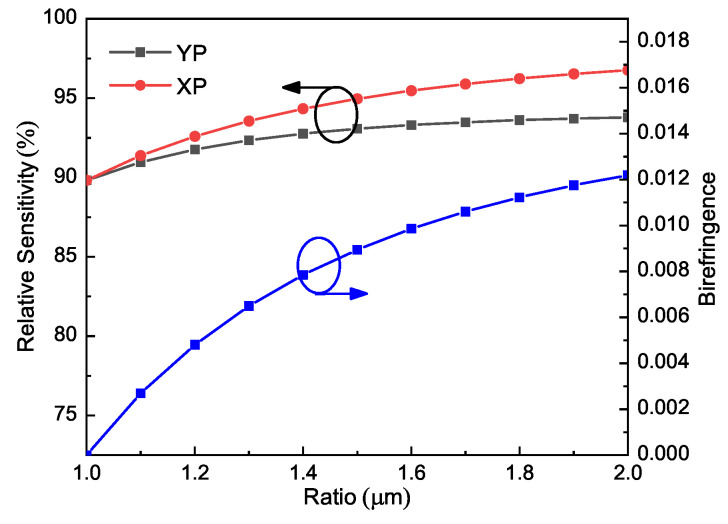
Relative sensitivity and birefringence of ethanol-filled PCF as functions of Ratio at 1 THz; the rest parameters are *W* = 280 μm, L=Ratio∗W, *d* = 10 μm.

To determine the optimal values of length:width, a parameter sweep of the ratio from 1 to 2 was conducted, while the rest of parameters were set as *W* = 280 μm, L=Ratio∗W, *d* = 10 μm. The sweep results are illustrated in Figure 4. It can be observed from the figure that both relative sensitivity and birefringence increase with the *Ratio*. In particular, the birefringence rises dramatically with the ratio. This is because a higher ratio means a more asymmetric core area, which in turn enhances the difference between the XP and YP modes. However, a higher ratio would increase the difficulty of fabrication as well. We therefore chose the ratio to be 1.5 as a compromise.

**Figure 5:**
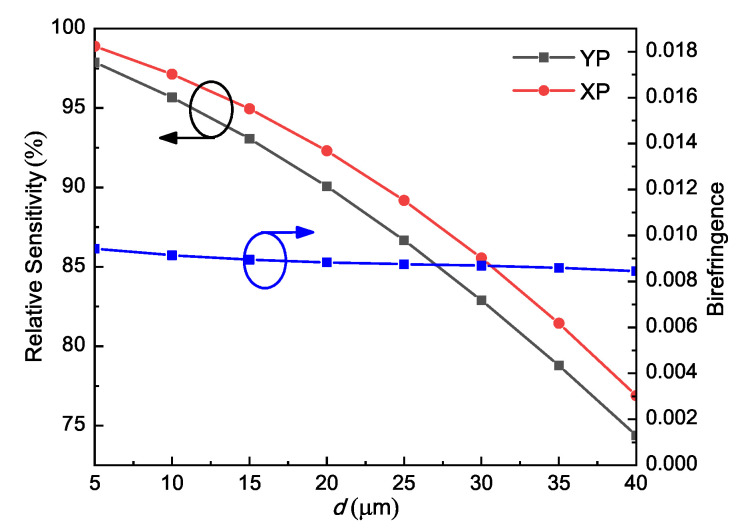
Relative sensitivity and birefringence of ethanol-filled PCF as functions of *d* at 1 THz; the other parameters are *W* = 280 μm, L=Ratio∗W, Ratio = 1.5.

Figure 5 shows the variations of the relative sensitivity and birefringence with the strut thickness *d* varying from 5 to 40 μm while other parameters were fixed at *W* = 280 μm, L=Ratio∗W, and Ratio = 1.5. According to the figure, the birefringence is quite stable and the relative sensitivity decreases rapidly when *d* increases. This is due to the fact that thicker struts can reduce the refractive index contrast between the core and cladding, thereby leading to a lower confinement ability of the PCF. As a result, a large portion of energy would be distributed in the struts rather than the core. Consequently, according to the Equations (1) and (2), the relative sensitivity was lower. Considering both the mechanical stability and sensing performance, we chose *d* = 10 μm as the optimal value.

**Figure 6:**
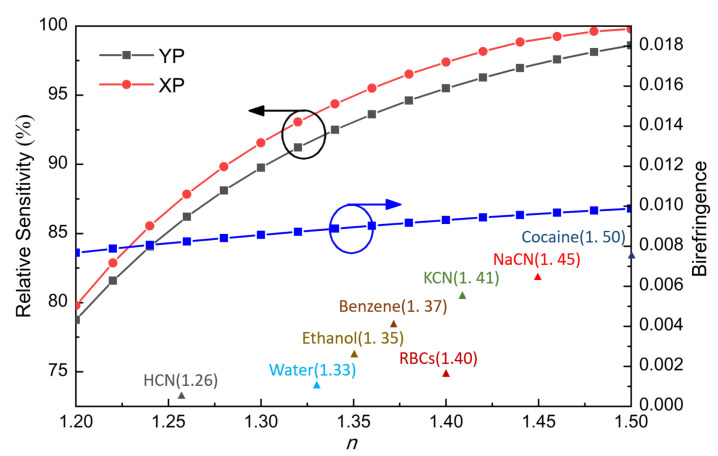
Relative sensitivity and birefringence of the proposed PCF as functions of refractive indices of the analytes at 1 THz.

After determining the optimal dimensions of the PCF, we then investigated the sensing performance of the proposed PCF for different analytes [21,22,24,33]. To evaluate the possibility of the PCF as a chemical sensor for wide scenarios, almost all the analytes that have been investigated in recent years were selected to be separately filled in the core area of our PCF. The resultant relative sensitivity of each analyte at 1 THz is shown in Figure 6. Different analytes with different refractive indices within the range from 1.2 to 1.5 are indicated.

As concluded from Figure 6, the lowest relative sensitivity (87.8%, XP mode) is obtained for the HCN case (*n* = 1.26) and the highest relative sensitivity (99.9%, XP mode) is achieved for the cocaine case (*n* = 1.50). The achieved birefringence is also reasonable, close to 0.01 for all the cases. Therefore, it can be concluded that this PCF has a high potential to sense many analytes within this refractive index range. Moreover, it is noticed from Figure 3, Figure 4, Figure 5 and Figure 6 that the relative sensitivity of the XP mode is always higher than that of the YP mode. It is probably because the XP mode has a smaller portion of energy distributed outside of the analyte-filling area, such as in strut and the air region in the cladding neighboring the core. In other words, light interacts more strongly with the XP mode. Therefore, the XP mode is chosen as the optimum mode for our PCF and only the performances of the XP mode will be displayed in the following.

**Figure 7:**
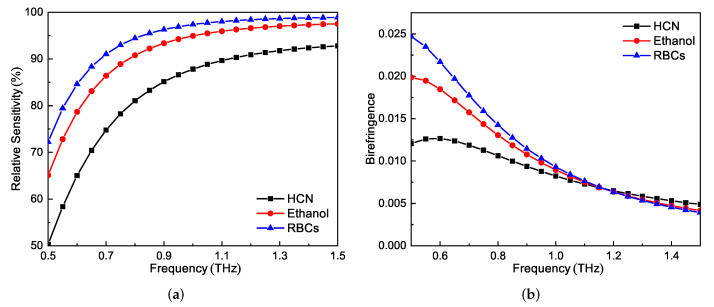
(**a**) Relative sensitivity and (**b**) birefringence of the proposed PCF as functions of frequency for the three different representative analytes.

To investigate other characteristics of the proposed PCF, three analytes with large refractive index gaps, i.e., HCN, ethanol, and RBCs, were selected as the representations. The relative sensitivity and birefringence as functions of frequency at optimal parameters are shown in Figure 7a,b. The sensitivity first increases rapidly, and then becomes relatively stable above 1 THz. On the other hand, the birefringence decreases as the frequency increases. The relative sensitivities of the three cases at 1 THz were 87.8%, 95.1%, and 97.4%, respectively. The corresponding birefringence values were 0.0082, 0.089, and 0.0093, respectively.

**Figure 8:**
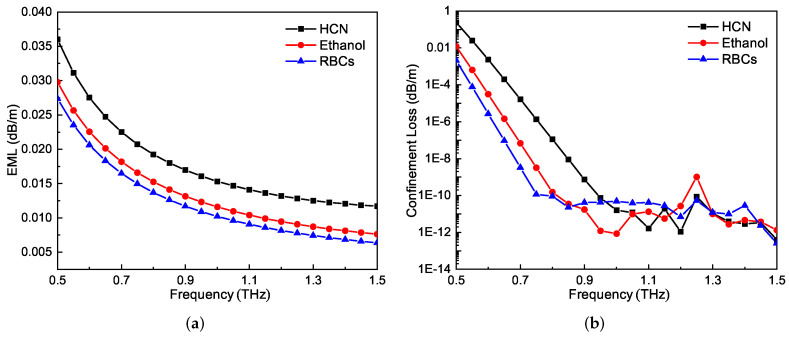
(**a**) Effective material absorption loss (EML) and (**b**) confinement loss of the proposed PCF as functions of frequency for the three three different representative analytes.

The loss properties of the PCF, including the EML and confinement loss, are shown in Figure 8a,b. With the increase of the frequency, both kinds of losses decrease. It should be noted that the confinement loss values below $10^{-12}$ cm${}^{-1}$ are unstable. This is because the values are too small and are comparable to the numerical errors introduced during the simulations. Compared to that of the EML, the confinement loss of the PCF is negligible. The total loss of the PCF can be as low as 0.01 cm${}^{-1}$ for the RBCs.

**Figure 9:**
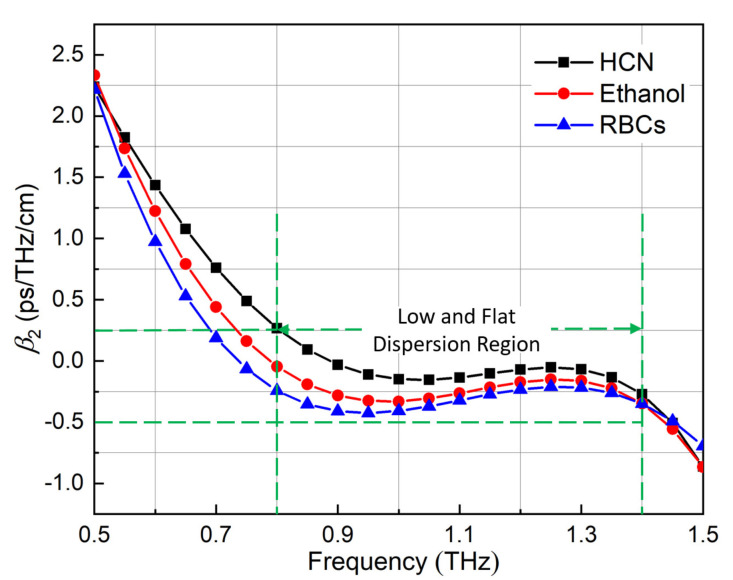
Dispersion performance of the proposed PCF as a function of frequency for the three different representative analytes.

The dispersion property of the proposed PCF for the three different representative analytes can be found in Figure 9. With the optimized parameters, the proposed PCF yielded a low and flat dispersion across a wide frequency band from 0.8 to 1.4 THz. Within this band, the dispersion of the HCN case was close to zero. The specific value was 0 ± 0.25 ps/THz/cm. The dispersions of the other two cases were more stable, −0.15 ± 0.15 ps/THz/cm and −0.32 ± 0.08 ps/THz/cm, respectively.

**Figure 10:**
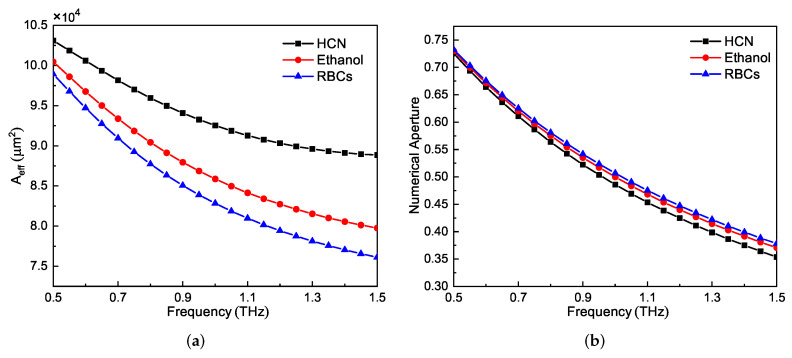
(**a**) $A_{eff}$ and (**b**) numerical aperture performances of the proposed PCF as functions of frequency for the three different representative analytes.

The performances of the effective mode areas of the proposed PCF for the three analytes as functions of frequency are shown in Figure 10a. The $A_{eff}$ values of the three cases over the entire frequency range are quite large. The specific values for HCN, ethanol, and RBC cases at 1 THz were 9.3 × 104 μm${}^{2}$, 8.6 × 104 μm${}^{2}$, and 8.3 × 104 μm${}^{2}$, respectively. The NA performance of the proposed PCF is shown in Figure 10b. The NA decreases as the frequency increases. At the selected central frequency, the specific NA values of the three cases were very high, 0.49, 0.50, and 0.51, respectively.

Besides, we compare the characteristics of our THz PCF-based sensor with the comparable state-of-the-art works in Table 1. The results of the ethanol case for this work, as an example, are shown in the table. It is noted that our design not only has the highest relative sensitivities, but also has higher birefringences, lower and flatter dispersions, lower losses, relatively larger effective mode areas, and higher numerical apertures, which together indicate the advantages of this work.

## 5. Discussion of Fabrication and Robustness

Although we did not fabricate and test a prototype of our design, we are quite confident that it can be realized with advanced fiber-fabrication techniques. Conventionally, to build a PCF, one needs to first construct a preform by stacking [1], drilling [34], or die extrusion [35], and then, draw the preform into a fiber through thermally-drawn process at an appropriate high temperature. In our case, due to the adoption of an asymmetrical structure, the stacking method is not suitable for implementation, as it can only be used to fabricate PCFs with circular air holes. Since the scale of the geometrical parameters is relatively large, the 3D printing method [36] is appropriate to print the whole structure directly. It is also worth noticing that rectangular and spider-web shaped PCFs have already been fabricated by Atakaraminas et al. [35] with the die extrusion method. Moreover, a variety of structural designs [37] and material compositions [38,39] have been realized successfully that are more complex than our design. Therefore, the implementation of the proposed PCF design should be successful with currently existing fabrication techniques.

**Figure 11:**
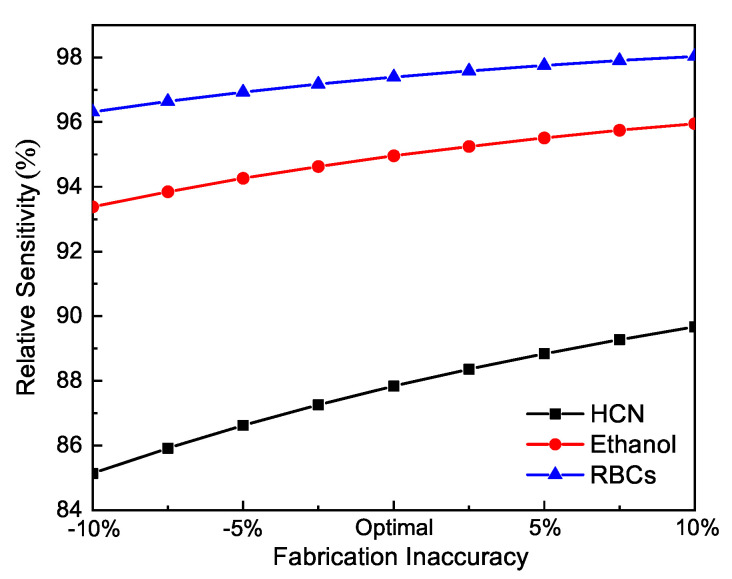
Relative sensitivity of the proposed PCF sensor as a function of the fabrication inaccuracy in the overall dimension.

In addition, we also considered the inaccuracy introduced during the fabrication process. The variation of the key performance with fluctuations of the overall size was analyzed to reveal the robustness of our design. Figure 11 shows the relative sensitivity as the dimensions of the entire structure vary by to ±10% from the optimal values, i.e., *W* = 280 μm, L=Ratio∗W, Ratio = 1.5, and *d* = 10 μm. Specifically, one can tune the whole structure by scaling *W* up or down, and other parameters would change proportionally. It can be observed from the figure that a ±10% variation in the whole structure only causes a maximum ±3% change of the relative sensitivity. Further investigations showed that a ±10% variation of the whole scale leads to moderate changes to other characteristics. For brevity, these figures are not included in this paper. It can be concluded that this design exhibits good tolerance of fabrication errors.

## 6. Conclusions

In this paper, a new rectangular suspended-core PCF with an extremely simple core and cladding has been developed in the THz regime for liquid sensing. Attributed to the hollow core, the light–liquid overlap is quite strong. Simulation results indicated that this Topas-based THz PCF yields ultra-high relative sensitivities (from 87.8% to 99.9%) and low losses for potentially all analytes with refractive indices ranging from 1.2 to 1.5 at 1.0 THz—i.e., eight analytes as examples were studied here. Specifically, three analytes were chosen to further investigate the performances of this design. The results show that high birefringences, low and flat dispersions, large effective model areas, and large numerical apertures can be obtained simultaneously by this PCF when the analytes are filled in the suspended core. Moreover, the potential fabrication techniques and the robustness of the proposed PCF were considered. Additionally, comparisons with previously reported liquid sensors demonstrated that this innovative fiber-based sensor has a much higher relative sensitivity and enhanced performance. It was conjectured that, with such great characteristics, this PCF sensor could serve as a great candidate in chemical sensing and biological sensing.

## Figures and Tables

**Table 1:** Comparisons between the proposed PCF sensor and comparable state-of-the-art sensors.

Ref.	Operate Region	Relative Sensitivity (%)	B	EML (cm${}^{-1}$)	$L_{c}$ (cm${}^{-1}$)	$\beta_{2}$ (ps/THz/cm)	$A_{\mathrm{eff}}$ (μm${}^{2}$)	NA
[11]	1.6 THz	85.7	0.005	-	1.7 $\times \;10^{-9}$	0.47 ± 0.265	7$\times 10^{4}$	0.37
[15]	1.0 THz	68.9	0.016	0.055	6.2 $\times \;10^{-12}$	1.07 ± 0.37	-	0.35
[21]	2.0 THz	85.8	0.009	0.023	1.6 $\times \;10^{-9}$	-	-	-
[22]	1.3 THz	78.8	-	-	2.2 $\times \;10^{-10}$	-	8 $\times \;10^{4}$	0.39
[23]	1.5 μm	63.4	0.00075	-	3.3 × $10^{-22}$	-	-	-
[24]	1.5 THz	80.9	-	-	1.1 $\times \;10^{-12}$	3.32 ± 1.82	17 $\times \;10^{4}$	-
[33]	1.0 THz	79.8	-	0.098	3.1 $\times \;10^{-13}$	-	-	-
This work	1.0 THz	95.1	0.009	0.012	1.0 $\times \;10^{-12}$	−0.15 ± 0.15	9 $\times \;10^{4}$	0.50

## Data Availability

Not applicable.
